# Care Needs and Clinical Outcomes of Older People with Dementia: A Population-Based Propensity Score-Matched Cohort Study

**DOI:** 10.1371/journal.pone.0124973

**Published:** 2015-05-08

**Authors:** Fei-Yuan Hsiao, Li-Ning Peng, Yu-Wen Wen, Chih-Kuang Liang, Pei-Ning Wang, Liang-Kung Chen

**Affiliations:** 1 Graduate Institute of Clinical Pharmacy, College of Medicine, National Taiwan University, Taipei, Taiwan; 2 School of Pharmacy, College of Medicine, National Taiwan University, Taipei, Taiwan; 3 Department of Pharmacy, National Taiwan University Hospital, Taipei, Taiwan; 4 Aging and Health Research Center, National Yang Ming University, Taipei, Taiwan; 5 Center for Geriatrics and Gerontology, Taipei Veterans General Hospital, Taipei, Taiwan; 6 Clinical Informatics and Medical Statistics Research Center, Chang Gung University, Taoyuan, Taiwan; 7 Geriatric Medicine Center, Kaohsiung Veterans General Hospital, Kaohsiung, Taiwan; 8 Department of Neurology, Taipei Veterans General Hospital, Taipei, Taiwan; Karolinska Institutet, ITALY

## Abstract

**Objective:**

To explore the healthcare resource utilization, psychotropic drug use and mortality of older people with dementia.

**Design:**

A nationwide propensity score-matched cohort study.

**Setting:**

National Health Insurance Research database.

**Participants:**

A total of 32,649 elderly people with dementia and their propensity-score matched controls (n=32,649).

**Measurements:**

Outpatient visits, inpatient care, psychotropic drug use, in-hospital mortality and all-cause mortality at 90 and 365 days.

**Results:**

Compared to the non-dementia group, a higher proportion of patients with dementia used inpatient services (1 year after index date: 20.91% vs. 9.55%), and the dementia group had more outpatient visits (median [standard deviation]: 7.00 [8.87] vs. 3.00 [8.30]). Furthermore, dementia cases with acute admission had the highest psychotropic drug utilization both at baseline and at the post-index dates (difference-in-differences: all <0.001). Dementia was associated with an increased risk of all-cause mortality (90 days, Odds ratio (OR)=1.85 [95%CI 1.67-2.05], p<0.001; 365 days, OR=1.59 [1.50-1.69], p<0.001) and in-hospital mortality (90 days, OR=1.97 [1.71-2.27], p<0.001; 365 days, OR=1.82 [1.61-2.05], p<0.001) compared to matched controls.

**Conclusions:**

When older people with dementia are admitted for acute illnesses, they may increase their use of psychotropic agents and their risk of death, particularly in-hospital mortality.

## Introduction

Dementia, a neurodegenerative disorder characterized by cognitive impairment, has attracted broad international attention due to its high prevalence and profound health effects in the elderly population.[1] The annual incidence of dementia is approximately 2–7% in people aged 65 years and older across all countries and is expected to increase sharply as populations age.[2] With the expected increase in people with dementia, various healthcare challenges related to dementia care may become important issues within the healthcare system. The majority of people with dementia go unrecognized when searching for healthcare services across the healthcare settings. Empirical data describing the healthcare utilization and clinical outcomes for these patients are scarce, and limitations in study design also limit clinical interpretations.

Although dementia has been reported to increase healthcare utilization and related medical costs, most studies were performed in the 1990s; these studies did not include modern healthcare patterns and were focused primarily on inpatient services.[3–6] The disease burden of dementia on the primary care sector remains unknown. This may limit the possibility for proper healthcare resource allocation because a large number of older people with dementia live in communities. Furthermore, studies have shown that the comorbidities of older people with dementia are significantly higher than those without dementia;[7] indeed, the increased hospital admissions of these patients may result from a failure of outpatient management of comorbidities rather than dementia *per se*.[8–11]

We know little about the utilization of specific drug categories, such as psychotropic agents (i.e., anxiolytics or antipsychotics) among demented people. This is concerning because behavioral and psychotic symptoms are common among people with dementia; these symptoms may be managed by pharmaceutical intervention.[12–15] According to The Alzheimer's Society, 77% of nurses who were surveyed reported treating people with dementia in the hospital setting with antipsychotic drugs[16] despite strong evidence of harmful adverse effects of these drugs. In particular, existing studies have raised significant concerns regarding the potential harms and adverse events associated with psychotropic drug use, such as dependency[17], road traffic accidents[18,19], accidental falls[20,21], fractures[22,23], or impaired cognitive function.[24–26] Furthermore, it remains unclear whether people with dementia are discharged with these medications, which may result in negative outcomes after potential long-term use.

The most significant knowledge gap regarding caring for people with dementia lies within the potential interactions between acute hospital admissions and dementia. In addition to the increased use of inpatient services among people with dementia, the long-term effects of acute hospital admissions and dementia care remain uninvestigated. When compared to people without dementia, people with dementia may have various healthcare needs during or after an acute admission. Moyle et al. suggested that the physical layout, particularly the busy environment, in hospital wards can be disorientating for people with dementia[27] and can trigger the occurrence of delirium. Kurrle et al. further reported that a period of hospitalization has an adverse effect on frail older people, particularly among people with dementia.[28] Without appropriate caution, people with dementia may suffer from more severe negative effects of hospitalizations than patients without dementia. However, there is a lack of empirical studies to test these hypotheses.

To overcome the above-mentioned knowledge gaps, the aim of this study was to evaluate healthcare utilizations, with a specific focus on medication use, in a cohort consisting of people with dementia and their propensity score-matched controls. Moreover, this study also aimed to explore the potential interaction between dementia and acute admission on the medication use and survival of people with dementia.

## Material and Methods

### Data sources

This is a population-based cohort study that utilized the National Health Insurance Research database (NHIRD). The NHIRD is a nationwide database consisting of anonymous eligibility and enrollment information as well as claims for visits, procedures, and prescription medications of more than 99% of the entire population in Taiwan (23 million). Individual patients are recorded as entering the NHIRD when they were covered by Taiwan’s mandatory National Health Insurance (NHI) program (from 1996), and individuals are recorded as leaving the program at death. For each visit, the NHIRD records the dates of the visits (outpatient visits, admissions and discharges) and up to 5 diagnoses coded by physicians according to the *International Classification of Disease*, *9th Edition* (ICD-9 CM codes).[29] The completeness and accuracy of the NHIRD are ensured by the Ministry of Health and Welfare and the National Health Insurance Administration of Taiwan. The database has been described in detail elsewhere[30] and has been a source for numerous epidemiological studies published in peer-reviewed journals.[31–33]

We used the 2001–2009 Longitudinal Health Insurance Database, which contains the claims data of two million beneficiaries (approximately 10% of the total population) randomly sampled from the NHIRD, as our data source. The age and gender distributions of the LHID are not significantly different from those of the original NHIRD cohort. The longitudinal nature of the LHID permits the identification of a cohort based upon diagnoses, health services, drug utilization, track medical history, and the establishment of a prescription drug profile and can determine the endpoint of drug treatment.

### Ethical statement

Because the identification numbers of all subjects in the NHRID were encrypted to protect individual privacy, this study was exempted from full review by the Institution Review Board of the National Taiwan University Hospital, and the requirement for informed consent was waived. The Institution Review Board of the National Taiwan University Hospital approved this study.

### Dementia cases and controls

From the LHID between January 1, 2001 and December 31, 2009, we identified people who were older than 65 years and who were first diagnosed with dementia, according to the following *International Classification of Diseases*, *Ninth Revision*, *Clinical Modification* (ICD-9-CM) codes: 290.0 (senile dementia, uncomplicated), 290.1× (presenile dementia), 290.2× (senile dementia with delusional or depressive features), 290.3× (senile dementia with delirium), and 290.4× (arteriosclerotic dementia). We did not include 331.0 (Alzheimer's disease) and 331.1 (frontotemporal dementia) to define dementia in our study because the prevalence of outpatient or inpatient visits when using these diagnostic codes was less than 5% in the NHIRD. To increase the identification of dementia cases, only those who had at least three outpatient or inpatient claim records of dementia-related diagnosis codes were selected as our dementia cases[34]. The date of the first diagnosis of dementia was designated as the index date for all identified dementia cases.

For each dementia case, a matched control was randomly selected from the NHIRD using the propensity score matching technique to account for baseline differences between the demented and non-demented groups.[35,36] The propensity score was estimated from a multivariable logistic regression model. Covariates included in the model were age, sex, and concomitant diseases (diabetes mellitus, hypertension, heart failure, stroke, transient ischemic attack, osteoporosis, osteoarthritis, chronic obstructive pulmonary disease and depression). This approach enables the determination of whether dementia was an independent predictor of outcomes.

### Outcome measures

The primary endpoint was the health-related resource use between the demented and non-demented groups. We focused on hospitalizations (number of admissions, length of stay, and primary diagnosis on admission) and outpatient services (number of visits and primary diagnosis on admission) during the one-year follow-up period after the index date. Additionally, we examined pharmacotherapies with known cognitive effects, including the use of antipsychotics (as defined by the Anatomical Therapeutic Chemical [ATC] classification system released by the WHO Collaborating Centre for Drug Statistics Methodology[37] [ATC code: N05A-, such as lithium, perphanazine, or fluphenazine]), anxiolytics (N05B-, such as hydroxyzine), hypnotics (benzodiazepine derivatives [N05CD-, such as estazolam] and z-drugs [N05CF-, such as zolpidem]), and antidepressants (N06A-, such as paroxetine or mirtazapine) one year before and after the index date. Based on this approach, most mood stabilizers (e.g., lithium) or drugs with anticholinergic effects (such as paroxetine or mirtazapine) were included in our measurement of “psychotropic drug” use. The secondary outcomes were all-cause mortality and in-hospital mortality at 90 and 365 days.

### Statistical analyses

McNemar tests were performed to compare categorical variables, and paired t-tests were used to compare continuous variables of both baseline characteristics as well as the health-related resource use. One pre-planned analysis was performed to further investigate the interactions between dementia and acute admission in relation to the use of psychotropic agents. The reason we mainly used pairwise comparisons was because of our "propensity score-matched cohort" study design. When outcomes were binary, propensity score methods allowed estimation of differences in proportions as most comorbid diseases were balanced between people with dementia and people without dementia in our study[38]. A difference-in-difference estimation was adopted to examine the difference in the use of psychotropic agents one year before and after the index date between the two study groups.[39] Conditional logistic regression models were used to examine the effects of dementia on 90-day and 365-day mortality (all-cause mortality, in-hospital mortality and in-hospital mortality associated with pneumonia) in our study subjects. All analyses were performed using SAS version 9.1.3 (SAS Institute Inc., Cary, NC, USA).

## Results

The study subjects consisted of 32,649 people with dementia and their propensity score-matched non-dementia controls (n = 32,649). The age and gender distributions in the dementia cases and controls were well matched. The dementia cases were slightly more likely to have a transient ischemic attack than the non-dementia group (dementia case: 2.27% vs. non-dementia control: 1.92%). In contrast, the dementia cases were slightly less likely to have hypertension (dementia case: 45.09% vs. non-dementia control: 42.70%) than the non-dementia group. Other comorbid diseases were balanced between the groups (Table 1).

**Table 1:** Dementia cases and their propensity-score matched cohort.

	Propensity-score matched cohort				
	(n = 51,384)				
	Dementia cases		Controls		
	(n = 25,692)		(n = 25,692)		
	n	%	n	%	p-value
Age group (in 5-yr increments)					0.0839
65–74	8021	31.22	8362	32.55	
75–84	12683	49.37	12797	49.81	
≥85	4988	19.41	4533	17.64	
Female	12418	48.33	12415	48.32	0.9899
Concomitant diseases					
Diabetes mellitus	5129	19.96	5345	20.80	0.0357
Hypertension	10971	42.70	11584	45.09	<0.0001
Heart failure	1225	4.77	1124	4.37	0.0391
Stroke	2796	10.88	2910	11.33	0.1347
Transient ischemic attack	582	2.27	493	1.92	0.0072
Osteoporosis	1552	6.04	1566	6.10	0.8159
Osteoarthritis	3926	15.28	4029	15.68	0.2528
COPD	1263	4.92	1191	4.64	0.1518
Depression	1538	5.99	1546	6.02	0.8997

Propensity score: age, gender, diabetes, hypertension, heart failure, stroke, transient ischemic attack, osteoporosis, osteoarthritis, COPD, depression

Compared to the non-dementia group, a higher proportion of patients with dementia used inpatient services (1 year after index date: 20.91% [7,829 admissions] vs. 9.55% [3,209 admissions]). For both groups, pneumonia was the most common condition for hospital admissions. Patients with dementia also had higher use of outpatient visits than the non-dementia group (1 year after the index date, number of outpatient visits, median [standard deviation] 7.00 [8.87] vs. 3.00 [8.30]). Dementia was the most common diagnosis for outpatient visits among the dementia group, while essential hypertension was the most common diagnosis for outpatient visits among non-dementia subjects (Table 2).

**Table 2:** Hospitalization and outpatient service utilization 1 year after the index date.

	Propensity-score matched cohort				
	(n = 51,384)				
	Dementia cases		Controls		
	(n = 25,692)		(n = 25,692)		
	n	%	n	%	p-value
**Hospitalizations**					
** Hospitalizations per individual**					<0.0001
0	20321	79.09	23239	90.45	
1	3694	14.38	1929	7.51	
2	1122	4.37	367	1.43	
3+	555	2.16	157	0.61	
**Total number of hospitalizations**	7829		3209		
**Length of stay**					<0.0001
Median [Std]	9.00	35.20	8.00	37.23	
< = 7	3414	43.61	1548	48.24	
8–30	3473	44.36	1276	39.76	
31–90	750	9.58	298	9.29	
91+	192	2.45	87	2.71	
**Primary diagnosis (Top 5)**					
	Pneumonia (ICD-9-CM: 486)		Pneumonia (ICD-9-CM: 486)		
	Other urethra and urinary tract disorders (599)		Other lung diseases (ICD-9-CM: 518)		
	Occlusion of cerebral arteries (ICD-9-CM: 434)		Other urethra and urinary tract disorders (599)		
	Other lung diseases (ICD-9-CM: 518)		Heart failure (ICD-9-CM: 428)		
	Dementia (ICD-9-CM: 290)		Chronic bronchitis (ICD-9-CM: 491)		
**Outpatient visits**					
**Outpatient visits per patient**					<0.0001
Median [Std]	7.00	8.87	3.00	8.30	
0	3685	14.34	9309	36.23	
1–5	6677	25.99	6281	24.45	
6–10	6165	24.00	4267	16.61	
11–15	4026	15.67	2657	10.34	
16+	5139	20.00	3178	12.37	
**Primary diagnosis (Top 5)**					
	Dementia (ICD-9-CM: 290)		Essential hypertension (ICD-9-CM: 401)		
	Diabetes mellitus (ICD-9-CM: 250)		Diabetes mellitus (ICD-9-CM: 250)		
	Essential hypertension (ICD-9-CM: 401)		Acute upper respiratory infections (465)		
	Occlusion of cerebral arteries (ICD-9-CM: 434)		Osteoarthrosis and associated disorders (715)		
	General symptom (ICD-9-CM: 780)		Hypertensive heart disease (ICD-9-CM: 402)		

We further divided our study subjects into two groups: those with and those without acute admission 1 year after the index date. Our results showed that subjects with dementia were more likely to be prescribed study medications, including antipsychotics, anxiolytics, hypnotics (benzodiazepine and z-drugs), and antidepressants. Furthermore, acute admissions had a significant effect on drug utilizations, particularly in subjects with dementia. Among these subjects, dementia cases with acute admissions had the highest drug utilization both at baseline and at the post-index dates (difference-in-differences: all <0.001). Among all of the drugs investigated, anxiolytics were the most frequently prescribed to our study subjects (dementia with acute admission: 30.12% [baseline] vs. 35.56% [post-index date]; control without acute admission: 10.74% [baseline] vs. 20.17% [post-index date]). Specifically, more than one-third (35.13%) of dementia cases with an acute admission were prescribed antipsychotics compared to only 4.79% in controls without acute admission (Table 3).

**Table 3:** Use of psychotropic agents 1 year before and after the index date, with or without acute admission.

	Dementia cases						Controls						
	(n = 25,692)						(n = 25,692)						
	w/ acute admission			w/o acute admission			w/ acute admission			w/o acute admission			
	n = 5,391			n = 20,321			n = 2,453			n = 23,239			
	(21.00%)			(79.00%)			(9.50%)			(90.50%)			
	n	%	Difference^a^	n	%	Difference^a^	n	%	Difference^a^	n	%	Difference^a^	Difference-in-difference^b^
**Drug utilization**													
Antipsychotics													
1 year before index date	729	13.57		1933	9.51		76	3.10		604	2.60		
1 year after index date	1887	35.13	+21.56%	5038	24.79	+15.28%	205	8.36	+5.26%	1114	4.79	+2.19%	<0.01
Anxiolytics													
1 year before index date	1618	30.12		5443	26.79		297	12.11		2496	10.74		
1 year after index date	1910	35.56	+5.44%	6086	29.95	+3.16%	687	28.01	+15.90%	4687	20.17	+9.43%	<0.01
Hypnotics, benzodiazepine													
1 year before index date	552	10.28		1677	8.25		84	3.42		700	3.01		
1 year after index date	854	15.90	+5.62%	2125	10.46	+2.21%	213	8.68	+5.26%	1232	5.30	+2.29%	<0.01
Hypnotics, z-drugs													
1 year before index date	731	13.61		2388	11.75		141	5.75		988	4.25		
1 year after index date	1136	21.15	+7.54%	3104	15.27	+3.52%	329	13.41	+7.66%	1677	7.22	+2.97%	<0.01
Antidepressants													
1 year before index date	673	12.53		2201	10.83		132	5.38		1067	4.59		
1 year after index date	1190	22.16	+9.63%	3858	18.99	+8.16%	263	10.72	+5.34%	1473	6.34	+1.75%	<0.01

Difference ^a^: The proportion of patients who used specific a drug category (1 year after index date-1 year before index date)

Difference-in-difference ^b^: The proportion of patients who used a specific drug category (1 year after index date-1 year before index date); dementia vs. control; after adjustment of the impact of acute admission

We found that dementia was associated with an increased risk of all-cause mortality (90 days, OR = 1.85 [95%CI 1.67–2.05], p<0.001; 365 days, OR = 1.59 [1.50–1.69], p<0.001) compared to matched controls. Moreover, dementia was associated with a two-fold increased risk of in-hospital mortality compared to matched controls (90 days, OR = 1.97 [1.71–2.27], p<0.001; 365 days, OR = 1.82 [1.61–2.05], p<0.001). Specifically, we found an increased risk of in-hospital mortality associated with pneumonia among dementia cases (90 days, OR = 1.62 [1.13–2.32], p = 0.01; 365 days, OR = 1.47 [1.08–2.00], p = 0.02) (Table 4).

**Table 4:** Conditional logistic regression on the risk of mortality.

	n		%	Odds Ratio (95% CI)		P
**90 days**						
**All-cause mortality**						
**Dementia**	1144	/25692	4.45	1.85	(1.67–2.05)	<0.0001
**Control**	678	/25692	2.64	1.00	(reference)	
**In-hospital mortality**						
**Dementia**	614	/25692	2.39	1.97	(1.71–2.27)	<0.0001
**Control**	339	/25692	1.32	1.00	(reference)	
**In-hospital mortality (pneumonia)**						
**Dementia**	86	/25692	0.33	1.62	(1.13–2.32)	0.01
**Control**	55	/25692	0.21	1.00	(reference)	
**365 days**						
**All-cause mortality**						
**Dementia**	3345	/25692	13.02	1.59	(1.50–1.69)	<0.0001
**Control**	2187	/25692	8.51	1.00	(reference)	
**In-hospital mortality**						
**Dementia**	799	/25692	3.11	1.82	(1.61–2.05)	<0.0001
**Control**	474	/25692	1.84	1.00	(reference)	
**In-hospital mortality (pneumonia)**						
**Dementia**	113	/25692	0.44	1.47	(1.08–2.00)	0.02
**Control**	78	/25692	0.30	1.00	(reference)	

## Discussion

Our study is the first to use the propensity score-matched cohort approach to quantify the healthcare utilization associated with dementia. Compared to previous studies, which only accounted for demographics (usually age and gender) or a few comorbid conditions, we provided credible estimates of the independent effects of dementia on healthcare utilization. Our most important finding was the identification of significant care needs for people with dementia who were discharged after an acute admission; these people had the highest use of psychotropic agents, both at baseline and at the post-index dates. Additionally, dementia was associated with an increased risk of death, particularly in-hospital mortality. Consistent with previous studies,[1,3–5] we found that the use of inpatient services by people with dementia was doubled when compared to non-dementia people. This may be because older people with dementia are more likely to have multiple comorbid conditions that place them at a higher risk to be hospitalized in general medical wards, which has been suggested in previous studies.[8] However, a recent review clearly reported the drawbacks and challenges of acute care for older people with dementia.[40] Older people with dementia are considered to be highly vulnerable to acute hospital admissions. It has been reported that disruption from the usual home routine may be the primary problem encountered by older people with dementia during acute hospital admissions.[41] Moreover, the hospital staff may be not experienced in dementia care, which may result in inappropriate physical and chemical restraints; additionally, this may result in unmet needs in such patients due to poor communication with older people with dementia.[42] Furthermore, clinicians may focus on acute care within their own specialties and ignore the potentially inappropriate medications for older people with dementia, which may lead to a worsening of their psychiatric symptoms.[27,43–45] Taken together, older people with dementia may be placed at a higher risk of adverse outcomes, which may occur during and after acute admission. This hypothesis is supported by our study because people with dementia had increased associated risks of in-hospital (all-cause and pneumonia-associated) and overall mortality.

However, there has been very little attention towards addressing and improving the quality of acute care for older people with dementia in acute care settings. Because people with dementia were usually admitted to acute hospitals for diseases other than dementia,[8] the greatest challenge in improving the quality of dementia care for older people is the management of the causes of acute admissions. In our study, the top five conditions of admissions among people with dementia were mainly preventable ambulatory care-sensitive conditions (ACSCs),[8] such as pneumonia and urinary tract infections. Early detection and management of acute illnesses in their early phase may minimize the need for hospitalizations. Furthermore, similar conditions were observed among non-demented people; therefore, ACSCs should be the main focus when caring for an aged population. Therefore, this may be considered as a universal approach in improving the quality of acute care for patients with and without dementia.

Our results raise a significant concern regarding the use of psychotropic agents among people with dementia. In particular, we observed an approximately three-fold increase in antipsychotic use one year after the diagnosis of dementia, which was independent of whether the patient suffered an acute admission event (with admission: 13.57% at baseline vs. 35.13% 1 year after diagnosis; without admission: 9.51% at baseline vs. 24.79% 1 year after diagnosis). People with dementia who experienced acute admissions after the diagnosis of dementia were more likely to be exposed to hypnotics (both benzodiazepine and z-hypnotics) compared to those without acute admissions after the diagnosis of dementia. The increased use of psychotropic agents during and after acute hospital admissions may also have resulted from the deterioration of the behavioral and psychotic symptoms of dementia, particularly when patients were unable to cope with unfriendly acute care environments.[27] This finding is supported by Banerjee, who indicated that physicians too often prescribe antipsychotics as a first-line response to managing the challenging behaviors of people with dementia.[46] These prescriptions may further result in admissions related to fractures or other associated adverse outcomes.[26] This finding also echoes the experience of caregivers who reported that the physical and mental conditions of demented people significantly deteriorated after acute hospital admissions.[41] Although non-pharmacological intervention should be prioritized in the management of behavioral and psychotic symptoms for dementia, these treatments are usually only available in specific units in acute hospitals.

Another safety concern regarding the higher utilization of antipsychotics among people with dementia than control is the risk of stroke. Previous studies have suggested that dementia itself could be an independent risk factor for stroke and the use of antipsychotics may further increase the risk of stroke in people with dementia.[47,48] Liu at al reported use of antipsychotics increased a 1.17-fold risk of stroke (hazard ratio 1.17, 95% CI 1.01–1.40; p<0.05) among people with dementia [47] while Imfeld et al reported a 4.5-fold increase of odds (OR 4.5, 95% CI 2.1–9.2) of transient ischemic attack for patients with Alzheimer disease treated with atypical antipsychotics.[48] Our study also found that people with dementia were slightly more likely to have a transient ischemic attack than the non-dementia group (dementia case: 2.27% vs. non-dementia control: 1.92%) and they had higher utilization of antipsychotics than control even before the index date.

The results of this study clearly demonstrate the long-term adverse outcomes in older people with dementia. Dementia itself was associated with an increased risk of all-cause, in-hospital and pneumonia-related in-hospital mortality. However, there is little information regarding the time of discharge to the end of life among older people with dementia. A study performed by Bradshaw et al. demonstrated that the outcomes for older people who were discharged from an acute hospital were very poor. More than half of the survivors did not recover their physical independence after an associated acute illness, and more than 70% of the survivors presented with significant behavioral and psychological symptoms.[49] This finding was also consistent with our findings regarding the sharp increased use of psychotropic agents among people with dementia and patients who experienced acute admissions. Therefore, more efforts should be placed on the long-term management of these people after they are discharged from an acute hospital.[50]

Despite the efforts in this study, there were several limitations. First, the dementia diagnosis in this study was determined using the claim data of NHIRD. As with all observational studies based on claim databases, we were not able to include variables not routinely captured in claim databases, such as social histories (cigarette or alcohol consumption), measurements of functional scales (e.g., MMSE), results of radiographs (e.g., CT/MRI) or laboratory tests. We were also not able to differentiate between dementia and other dementia-like etiologies. Nevertheless, we adopted a published algorithm to increase the identification of dementia cases. Additionally, we adopted a “newly diagnosed dementia cohort” study design to attenuate the potential effect of the severity of dementia. Second, we could not confirm whether the patients had adhered to the instructions for taking their prescriptions of psychotropic medications. However, this is a common challenge in most studies. Third, indications for the increased use of psychotropic agents were unknown from the claim data, such that we were not able to presume that these prescriptions were given as chemical restraints. Nevertheless, there is a generalized consensus that psychotropic agents should be terminated whenever possible. Therefore, the rising trends of psychotropic agent use for older patients with and without dementia in this study deserve further attention.

## Conclusion

Our study suggested that specific attention should be paid when older people with dementia are admitted to general medical wards. A few models have been developed to improve the quality of acute care for older people with dementia, including a special unit or shared-care model. However, improving the awareness of hospital staff regarding dementia is of great importance because the negative effect of acute care on older people with dementia may only be the tip of the iceberg. The cognitive impairments in elderly patients admitted to general medical wards may be overlooked by hospital staff, which may lead to a significant functional decline in these patients. Taken together, improving the awareness and understanding of dementia among general hospital staff is important in order to optimize the care for older people with dementia. The quality of dementia care in general hospitals has been regarded as an important indicator of the quality of acute care in general hospitals.
